# Artificial Intelligence-Driven Prediction Modeling and Decision Making in Spine Surgery Using Hybrid Machine Learning Models

**DOI:** 10.3390/jpm12040509

**Published:** 2022-03-22

**Authors:** Babak Saravi, Frank Hassel, Sara Ülkümen, Alisia Zink, Veronika Shavlokhova, Sebastien Couillard-Despres, Martin Boeker, Peter Obid, Gernot Michael Lang

**Affiliations:** 1 Department of Orthopedics and Trauma Surgery, Medical Center-University of Freiburg, Faculty of Medicine, University of Freiburg, 79108 Freiburg, Germany; sara.uelkuemen@hotmail.de (S.Ü.); pm.obid@gmail.com (P.O.); gernotmichaellang@gmail.com (G.M.L.); 2 Department of Spine Surgery, Loretto Hospital, 79100 Freiburg, Germany; frank.hassel@rkk-klinikum.de (F.H.); alisia.zink@gmail.com (A.Z.); 3 Institute of Experimental Neuroregeneration, Spinal Cord Injury and Tissue Regeneration Center Salzburg (SCI-TReCS), Paracelsus Medical University, 5020 Salzburg, Austria; s.couillard-despres@pmu.ac.at; 4 Department of Oral and Maxillofacial Surgery, University Hospital Heidelberg, 69120 Heidelberg, Germany; veronika.shavlokhova@med.uni-heidelberg.de; 5 Austrian Cluster for Tissue Regeneration, 1200 Vienna, Austria; 6 Intelligence and Informatics in Medicine, Medical Center Rechts der Isar, School of Medicine, Technical University of Munich, 81675 Munich, Germany; martin.boeker@tum.de

**Keywords:** mixed data, deep learning, deep neural networks, hybrid networks, multi-input, artificial intelligence, prediction, healthcare, machine learning, spine, degeneration

## Abstract

Healthcare systems worldwide generate vast amounts of data from many different sources. Although of high complexity for a human being, it is essential to determine the patterns and minor variations in the genomic, radiological, laboratory, or clinical data that reliably differentiate phenotypes or allow high predictive accuracy in health-related tasks. Convolutional neural networks (CNN) are increasingly applied to image data for various tasks. Its use for non-imaging data becomes feasible through different modern machine learning techniques, converting non-imaging data into images before inputting them into the CNN model. Considering also that healthcare providers do not solely use one data modality for their decisions, this approach opens the door for multi-input/mixed data models which use a combination of patient information, such as genomic, radiological, and clinical data, to train a hybrid deep learning model. Thus, this reflects the main characteristic of artificial intelligence: simulating natural human behavior. The present review focuses on key advances in machine and deep learning, allowing for multi-perspective pattern recognition across the entire information set of patients in spine surgery. This is the first review of artificial intelligence focusing on hybrid models for deep learning applications in spine surgery, to the best of our knowledge. This is especially interesting as future tools are unlikely to use solely one data modality. The techniques discussed could become important in establishing a new approach to decision-making in spine surgery based on three fundamental pillars: (1) patient-specific, (2) artificial intelligence-driven, (3) integrating multimodal data. The findings reveal promising research that already took place to develop multi-input mixed-data hybrid decision-supporting models. Their implementation in spine surgery may hence be only a matter of time.

## 1. Introduction

Low back pain is one of the most frequently observed clinical conditions, and degenerative spine disease seems to be a leading driver of low back pain [1]. The global prevalence of low back pain increased from 377.5 million in 1990 to 577.0 million in 2017 [2]. The years lived with a disability increased globally from 42.5 million in 1990 to 64.9 million in 2017, representing an increase of 52.7%. Degenerative spinal disease is a common and impairing condition resulting in high socio-economic costs. Direct medical expenses spent on low-back pain doubled to 102 billion USD between 1997 and 2005, and the number of lumbar fusion procedures has quadrupled over the past 20 years, resulting in significantly increased healthcare costs [2].

Interestingly, the increase in performed surgeries is not directly proportional to improved patient outcomes. Impaired quality of life, persistent pain, and functional problems are reported in up to 40% of patients undergoing low back pain surgery and 20–24% undergoing revision surgeries [3,4]. Indications influencing the decision as to whether a patient should undergo surgery are not entirely based on guidelines but rather on discussions between the surgeon and patient, as well as the expertise and skills of the surgeon. Furthermore, there are no clear guidelines on surgical techniques for treating degenerative spinal diseases; as such, it remains unclear as to whether one treatment approach might perform better in particular cases than another. Overall, there is a considerable lack of data-driven decision-making in low back pain patients, which is particularly concerning when considering the global burden associated with low back pain.

Medical healthcare is driven by an incredible increase in the amount of data generated through various diagnostic tools and nodes within the healthcare systems. Patient data are the fundaments healthcare providers use to find the best fitting prognosis and diagnosis for each patient. Decisions are based on patterns across these datasets that guide towards the “right” diagnosis. Moreover, prognosis healthcare providers utilize these datasets to justify a specific treatment approach. Therefore, the correct interpretation of these datasets is crucial and directly impacts patient outcomes and the operations of healthcare systems.

Furthermore, improvements in treatment guidelines are mainly based on research that has been performed on such datasets. Researchers using these data might not be aware of the patterns hidden in their collected datasets. The process of finding patterns in large datasets which specifically fall under the category of big-data research is called data mining [5]. However, oftentimes, clinical researchers might not have profound knowledge in (bio)statistics personally, nor access to biostatisticians, to apply the best available tools to their datasets to extract all relevant pieces of information. Therefore, it is of high relevance that such datasets are made public and anonymized so that data scientists can use them and possibly determine these patterns using modern data-mining technologies.

The term “digital health” stands for the digitalization of healthcare data that was previously only assessed in an unproductive way through paper-based forms. New healthcare applications have become increasingly relevant and available. Such applications can range from mobile health applications, consumer techs, and telehealth for monitoring and guiding patients to precision medicine utilizing patient-specific data in artificial intelligence and bioinformatics models for individualized treatment approaches.

Machine learning is a subset of artificial intelligence and refers to computer techniques that allow complex tasks to be solved in a reproducible and standardized way. Machine learning combines biostatistics, mathematics, and computer science into one problem-solving pathway. One advantage is its efficiency and effectiveness, as the underlying programming code can be modified to enhance the accuracy of paths that solve a specific task. In this way, it can be more controllable, cost-efficient, and less error-prone than its “human template.” Although the number of publications and citations in artificial-intelligence-related papers on healthcare topics is overwhelming, the technique is still at the beginning of its maturity. The industry highly supports the progress because of the great potential to improve medical research and clinical care, particularly as healthcare providers increasingly establish electronic health records in their institutions.

Predictive analysis with classical statistical techniques, such as regression models, applied on these datasets has been the gold standard to date. One may ask about the advantages of advanced machine learning techniques over simple regression analysis using widely available statistical software for predictive analysis. It is hard to draw a distinct line indicating where basic statistical methods end and machine learning begins. It is often debated whether statistical techniques are somehow also considered as machine learning techniques, as in these cases, computers are using mathematical models to test a specific hypothesis. The primary differentiation might be the purpose of the application. Statistical methods, such as regression models, aim to find associations between independent variables (e.g., age, sex, body mass index) and dependent variables (e.g., patient-related outcome measures). Contrastingly, machine learning models also use statistical methods but aim to learn from training datasets, helping them to make more accurate predictions on the validation dataset so that the model can be reliably used on other independent datasets for predictive analysis. Hence, machine learning could be explained as focusing on predictive results, whereas simple statistical models analyze significant relationships. However, one further differentiation might be interpretability. The more complex a machine learning technique gets, the more accurate it can become at the cost of interpretability. For example, the lasso regression is a machine learning technique using regression analysis for feature selection and prediction. It has the advantage that it is not necessary to find the relevant independent variables first, as is required in linear regression modes. Its application is quite simple, and the interpretability is high. In contrast, deep learning, which is a subgroup of machine learning and will be discussed later, can get very complex but also very accurate; however, this comes at the cost of interpretability. The general principles of machine learning discussed in this review might help to differentiate between the most utilized approaches.

One significant barrier of machine learning applications is that reliable learning processes are very data-hungry. Machine learning is highly dependent on the premise that a large dataset is available. As computers cannot process visual and textual information the way human brains do, the algorithm needs to know what it is predicting or classifying in order to make decisions. When classification tasks need to be solved or specific areas need to be predicted, annotations are necessary. Data annotations help to make the input data understandable for computers. The task of the data scientist is to reliably label data such as text, audio, images, and video so it can be recognized by machine learning models and used to solve prediction and classification tasks. However, this process can be highly time-consuming, which might represent a major flaw in the implementation of machine learning algorithms. Non-accurate labeling will ultimately lead to inaccurate problem-solving. In previous research entitled “Deep Learning: A Critical Appraisal” [4], Marcus et al. proposed ten concerns associated with machine learning research, and data hungriness was listed as the top factor. He noted that “in problems where data are limited, deep learning often is not an ideal solution” [6]. Data-hungriness was also considered an unsolved problem in artificial intelligence (AI) research, described in Martin Ford’s book “Architects of Intelligence: the Truth About AI From the People Building It” [7]. Most of the researchers interviewed in his book encourage the development of more data-efficient algorithms. Four pillars relevant for the implementation and interpretation of machine learning algorithms were described by Cutillo et al. based on discussions at the National Institute of Health (NIH) healthcare workshop in 2019 [8]. These were Trustworthiness, Explainability, Usability, Transparency, and Fairness.

An increase in data efficiency cannot be made feasible only by increasing the number of input samples but also by improving the machine learning architecture itself. One way to do this is to consider that different data types might contribute differently to the problem-solving task and that the connection between data types might also be relevant. Discussing the data dependency of machine learning algorithms and different hybrid models capable of processing different data types is, unfortunately, a research field that has not received the necessary attention yet. In particular, the translation of such hybrid algorithms to a clinical environment with real-world applications has not yet been reviewed. Our workgroup is currently investigating novel hybrid machine learning algorithms for applications in spine surgery. The search for comparable models in the literature while building the architecture revealed an unexpected lack of research in this field. This review discusses hybrid algorithms for multimodal data processing and their implementation in spine research to close the gap currently available in the literature. The findings could help healthcare stakeholders plan and implement these promising algorithms in clinics.

## 2. Relevance of Digitalization in Spine Surgery

### 2.1. The Need for Structured Decision Making in Spine Surgery

A step towards precision and data-driven spine surgery can be achieved by meeting the significant requirement of developing informative outcomes in assessments. These include regular outcome assessments of patients, preferably utilizing digital app-based assessment forms, and the necessity to implement these outcomes as dependent variables in future risk assessment tools. Notably, the improvement of patient-related outcome measures (PROMs) should be the primary goal of decision-making. The value of such outcome measures is more critical in clinics than surrogate markers such as laboratory markers and classical clinical variables such as revision surgery, readmission, or absence of surgical infections. Our previous research has shown that patient-related outcome measures do not necessarily correlate with the factors a surgeon might consider relevant. For example, we could show that patient-related outcome measures were more correlated with the length of hospital stay than with postoperative complication rates [9]. Therefore, patient-related outcome measures should be an integral part of every predictive tool in spine surgery. In spine surgery, commonly utilized patient-related outcome measures include the Oswestry Disability Index, Core Outcome Measure Index (COMI), the eq-5D, SF-36 form, Numeric Rating Scale of pain, and the Visual Analogue Scale of pain, in addition to others [10]. Notably, Breakwell et al. reported in their publication entitled “Should we all go to the PROM? The first two years of the British Spine Registry” that a significant amount of PROMs forms were entered by the patients themselves [11]. Hence, an app-based tool transferring the results from the PROMs to a central database could be more time-efficient for spine surgeons. An additional benefit would be that outcomes could be compared considering all contributing institutes, and necessary quality-control steps could be performed in an early phase. This could also be very cost-efficient for healthcare institutes.

The integration of these patient-related outcome measures as dependent variables in clinical decision support tools would allow outcomes to be predicted during prospective follow-ups based on a set of several textual independent variables such as surgical technique, preoperative markers, as well as other data modalities such as imaging. This approach could reliably analyze large volumes of data based on previous data input and suggest next steps for treatment, flag potential problems, and enhance care team efficiency. Furthermore, this PROMs-including approach allows surgeons to discuss the possible outcome with patients and therefore improves the communication with patients. Contrastingly, a communication style in which the surgeon advises against surgery based on his subjective experience might lead to a negative surgeon-patient relationship. Such data-driven support tools might also be better to help surgeons communicate with patients.

### 2.2. Database Repositories for Machine Learning Applications in Spine Surgery

Databases are repositing data for future research. They are dedicated to housing data related to scientific research on a platform that can be restricted for access or publicly available. One often-used approach is to limit access to all contributors of the database. In this way, the database integrates a simple reward system: the contribution of data allows contributors to use the gathered data. Databases can collect and store a heterogeneous set of patient data and large datasets that fall under the category of big data. Usually, data in online medical databases are stored anonymously, maintaining that data cannot be linked to the patients’ personal information. In such cases, radiological images can be stored with genetic and clinical data, all having a unique identification number linking the different datatypes of the case. These databases cover a wide range of data, including those related to cancer research, disease burden, nutrition and health, and genetics and the environment. Researchers can apply for access to data based on the scope of the database and the application procedures required to perform relevant medical research.

**Figure 1:**
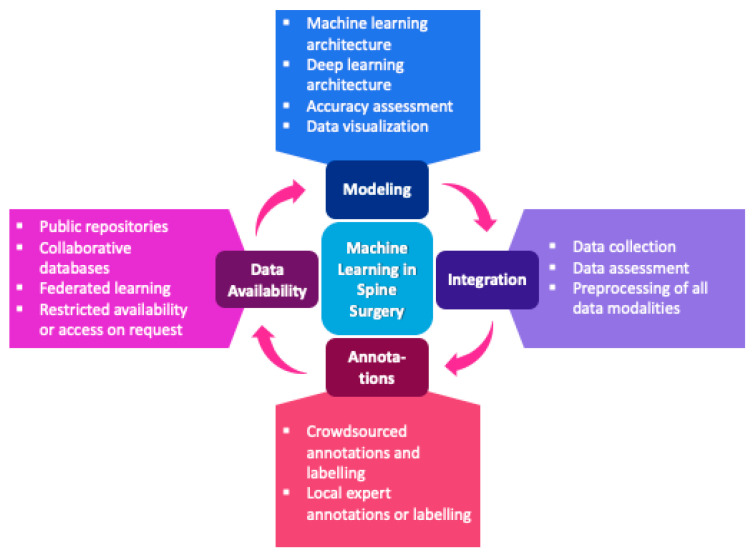
Workflow of machine learning applications in spine surgery.

For machine learning purposes, these data can also be labeled/annotated before being uploaded, allowing for utilization via data scientists. Although impactful machine learning models published to date might deal with a well-annotated dataset, the annotation process requires the necessary infrastructure, expertise, and resources, as it is very time-consuming depending on the number of data points. Considering the complexity of data annotation, crowdsourcing platforms are currently emerging. In this crowdsourcing model, the data are annotated by multiple crowdsourcing workers. One advantage is that the labeling can be checked against the consensus label using statistical parameters such as the inter-annotator agreement. Furthermore, this approach could lead to a more generalizable annotation style within the dataset. Therefore, the model might better predict future datasets coming from other workgroups. Crowdsourcing applications introduced were, for example, applications to database curation, the identification of medical terms in patient-authored texts, and the diagnosis of diseases from medical images [12,13,14]. Such platforms could also be applied by institutes in spine surgery. Although recent studies have shown that the accuracy performed by crowd workers is mainly similar to the individual annotation considering a given task, crowdsourcing is more resource-oriented and reliable [15,16,17]. The workflow of machine learning applications in spine surgery is shown in Figure 1.

Several databases are available that house an impressive number of global biomedical data. Notably, these repositories are regularly updated and extended using new image sets and data types provided by multiple institutions. Thus, they are often used by machine learning research studies, which is essential for progress in the field and exemplary for upcoming databases. For example, the GDC data portal [18] can provide RNA-sequencing, whole-genome, whole-exome sequencing, targeted sequencing, genotype, tissue and diagnostic slides, and ATAC-seq data. These data types could also be used as an input in hybrid machine learning models along with imaging and clinical data types to solve prediction tasks related to spinal oncology. Access to these platforms can be obtained from researchers but only for subsets of the whole dataset. The general principle of these platforms is that only data that will be used can be extracted. However, all mentioned databases do not contain data labeling and annotations. Considering that there can be vast amounts of data depending on the research question, this might be a significant limitation for using these data for machine learning purposes. Several sources of public databases are accessible by anyone who wants to train and test their machine learning models. One such example is the Kaggle Dataset collection, which contains several algorithms and datasets in spine surgery [19,20]. These datasets are often used for competitions and training novel machine learning methods to determine whether they outperform existing models. This allows for a peer-review process as the algorithms are publicly available and commented on by other data scientists, validating the algorithm on the dataset provided and external datasets. However, since journal peer-reviewers may not have the resources to retest provided datasets with the algorithm code, often uploaded in GitHub repositories [21], such open peer-review processes meet crucial research goals, including validity, objectivity, and reliability. Furthermore, provided datasets and codes from the workgroups may not be available after some time. This represents a significant flaw in the assessment and development process of machine learning algorithms for healthcare applications. Publications are not the only relevant output of research; research data should also be considered. This is particularly true when considering that more accurate analysis pathways might not have developed when the study was conducted. This paradigm led to the emergence of data journals, such as Scientific Data from Nature [22] or GigaScience from Oxford Academic [23] in which the data can remain available for future analysis and validity assessments.

Notably, in surgical fields, such databases are still scarce. One of the largest and most intuitive databases in orthopedic surgery is the Osteoarthritis Initiative (OAI) database [24] from the National Institute of Health, which includes ten-year multi-center observational data of knee osteoarthritis cases. It includes DICOM images, clinical data, and laboratory data, and it is one of the few and most extensive repositories in orthopedic surgery capable of integrating multimodal data. Unfortunately, to the best of our knowledge, the only database that seems to include multimodal data in spine surgery is the Austrian Spinal Cord Injury Study [25]. The database contains longitudinal data on spinal cord injury cases in Austria and includes clinical data with patient-related outcome measures and imaging data. Other databases in spine surgery, which mainly include tabular clinical data, are the American College of Surgeon National Surgical Quality Improvement Project (ASC-NSQIP) database, the National Inpatient Sample (NIS) database, the Medicare and Private Insurance Database, the American Spine Registry, and the British Spine Registry [11,26,27]. The SORG (“Sorg Orthopaedic Research Group”) has introduced the most recognized and cited predictive machine learning models, which can be accessed for free on their website [28]. They were already externally validated several times and include mortality prediction algorithms in spinal oncology, PROMs, and postoperative opioid use predictions after spine surgery, as well as discharge disposition for lumbar spinal surgery. Validation and external validation studies are both accessible on the website.

Another emerging field aiming to address the data handling problem in machine learning is privacy-first federated learning [29]. Federated learning [30,31] aims to train machine learning algorithms collaboratively without the need to transfer medical datasets. This approach would address the data governance and privacy politics, often limiting the use of medical data depending on the country where the research is conducted. Federated learning was extensively applied in mobile and edge device applications and is currently increasingly applied in healthcare environments [32,33]. It enables the assessment and development of models collaboratively using peer-review techniques without transferring the medical data out of the institutions where the data were obtained. Instead, machine learning training and testing take place on an institutional level, and only model architecture information and parameters are transferred between the collaborators. Recent studies have shown that machine learning models trained by Federated Learning can achieve similar accuracies to models that were implemented using central databases and are even superior to those processed on a single-institution-level [34,35]. Successful implementation of Federated Learning approaches could thus hold significant potential for enabling resource-oriented precision healthcare at a large scale, with external validation to overcome selection bias in model parameters and to promote the optimal processing of patients’ data by respecting the necessary governance and privacy policies of the participants [33]. Nevertheless, this approach still requires essential infrastructure and quality management processes to ensure that the applications perform well and do not impair healthcare processes or violate patient privacy rules. 

Despite the advantages of Federated Learning, this method still has some disadvantages. For example, as described above, the integration of medical datasets in public databases could lead to more extensive research, and the investigation would not be limited to the collaborators. Furthermore, successful model training still depends on factors such as data labeling, data quality, bias, and standardization [36]. These issues would be better targeted when databases are accessible by more researchers and crowdfunding workers dealing with data annotation. This would be the case for both Federated and non-Federated Learning techniques. Appropriate protocols would be required, focusing on well-designed studies, standardized data extractions, standardized labeling and annotation of data, accuracy assessments and quality management, and regularly updated techniques to assess bias or failures. Considering this, Federated Learning would be a feasible approach to overcome data transfer limitations between institutions.

## 3. Hybrid Machine Learning Models for Classification and Prediction Tasks

### 3.1. Textual Data Conversion Methods for Deep Learning Approaches

Deep learning is a subset of machine learning utilizing artificial neural networks for information processing. Artificial neural networks were applied in various fields, including image analysis, natural language processing, and video or speech recognition [37]. The technique is based on information processing, similar to how humans process visual inputs. Artificial neurons are the units of the network, are organized in layers, and transmit the information from layer to layer depending on the predefined neural network architecture. Receptive fields, convolution kernels, and hierarchical feature abstraction are used in multiple layers to analyze data between the input and output layers [38]. The technique is used to process information with spatial or temporal dependencies, such as images where the spatial arrangement of pixels contains information about each image’s content. Image processing through network layers is performed whereby the flow through the layers that are applying different mathematical functions leads to the desired pattern recognition. The architectures can be very heterogeneous, and the model is built depending on the task that needs to be solved. In healthcare data processing, these models are usually feedforward architectures where the information is processed from the input to the output layer. Tasks can, for example, be solved through classification or multi-classification models (i.e., for predicting categorical variables such as disease and health) or regression models (i.e., for predicting a continuous variable, such as a score) [39].

Deep neural networks have several benefits: finding hidden structures in the provided data, data augmentation, feature extraction, dimension reduction, optimum action selection in time-series data, and semi-supervised learning, including non-labeled data [40]. These neural networks are supposed to process imaging data; however, a large amount of healthcare data are textual (e.g., laboratory data, clinical data, genomic data). One way of using the strengths and advantages of convolutional neural networks to process such information is to rearrange tabular data into a 2-D shape that considers the relationship between the feature variables. Feature variables, in this case, are all variables that might be relevant for the task that has to be solved (e.g., outcome score prediction). To the best of our knowledge, four methods have been proposed for transforming non-image data into images shaped for use in convolutional neural networks. All transform the feature vector to a feature matrix using different transformation schemes. Ma et al. presented OmicsMapNet, a conversion method that arranges features based on annotations [41]. Hence, OmicsMapNet could be applied to RNA-Seq expression data of TCGA glioma samples. The functional hierarchical structure of the genes based on treemaps was used to construct 2-D images considering the function of genes. Consequently, gene functions could be learned for future functional analysis of independent RNA-seq datasets [41]. The implementation of such conversions and inclusions of RNA-Seq data within hybrid models could allow researchers to analyze survival parameters (such as mortality or time to recurrence) in spinal oncology and better plan the follow-up of patients.

The second method was published by Sharma et al. in 2019 to convert genetic data and other textual data into images [40]. Their DeepInsight method uses similarity measurement or linear dimensionality reduction techniques, such as t-SNE or kernel principal component analysis, for data transformation [42]. The advantage of DeepInsight is that it can construct images where similar features are put together in neighboring elements, which is proposed to be beneficial for data processing through the convolutional neural network [40]. Another method was described by Baszir et al. in 2020, who introduced REFINED, which also considers similarities between features to generate 2-D feature maps in which the distance is reduced utilizing the Bayesian Metric Multidimensional Scaling Approach [43]. Both methods could have particular relevance for the analyses of gene phenotypes in spine research. For example, there is increasing evidence suggesting that there are genetic architectures of low back pain [44]. The use of more precise algorithms could help to classify patients according to their genetic architecture; in doing so, surgeons could plan therapeutical management according to the principles of precision medicine.

Recently, Kanber et al. [45] published another approach that compared five different conversion schemes for transforming sparse data into structured image sets. The transformation either applied for linear filling order strategies, keeping the initial ordering of the spatial feature intact (ASIS), random ordering with a randomized ordering of spatial features (RAND), and linear (SDIC) or circular (SDIC_c) orderings combined with mathematical calculations using the Pearson product-moment correlation coefficients to construct the image sets. They then compared the accuracy of the CNN application on the converted images to Random Forest decision tree classifier and the DeepInsight transformation scheme introduced by Sharma et al. [40] The highest accuracy of all mentioned transformation schemes was achieved by SDIC when applied to two public databases of textual data. The algorithm could be used to convert clinical information and surgery-specific parameters (such as operation time) into 2D shaped images which can be applied in deep learning models. This broadens the number of possible methods that could be applied to the initial dataset and allows for comparison between multiple techniques to obtain more precise prediction models in spine surgery clinics. Other published methods might not transform the textual data into images first. Still, they process textual information with imaging data in different ways to pass the layers of the CNN for classification or regression tasks. For example, the Dynamic Affine Feature Map Transform (DAFT), published by Pölsterl et al. in 2021, fuses information of high-dimensionality 3D MRI images and tabular data without first combining converted tabular features and images before feeding them into the network [46]. Specifically, DAFT rescales and shifts the feature maps of a convolutional layer conditional on a patients’ tabular data in the CNN. Especially when considering the complexity and resource intensity of 3D images used in spine surgery (MRI, CT), this approach helps to effectively process such large datasets for prediction tests. Other published techniques were the processing of microscopic images, clinical data, and genomic data by Hao et al. (PAGE-Net) for survival analysis [47], and the processing of histological images and genomic biomarkers by Mobadersany et al. [48] Several other authors also used a multilayer perceptron (MLP) prior to the concatenation of image and tabular data before processing the information in the CNN (“early concatenation”) [49,50,51]. The use of histological images and genomic biomarkers analyzed by these techniques could be routinely implemented in spine clinics. For example, blood from patients is usually taken for analysis before and after surgery on a routine basis. Meanwhile, the tissue taken in disc herniation surgery, for example, is considered biological waste and discarded. When more effective, cost-efficient, and fast diagnostic methods, such as in vivo reflectance confocal microscopy [52], are available in the future, the prediction could be made intraoperatively, and further management can be translated directly from the available data sets of each patient (“precision medicine”). Overall, the extensive amount of research that has been performed within the last few years indicates the possibility of reliable textual data processing for multimodal hybrid deep learning models.

### 3.2. Multi-Input Mixed Data Deep Learning Models

**Figure 2:**
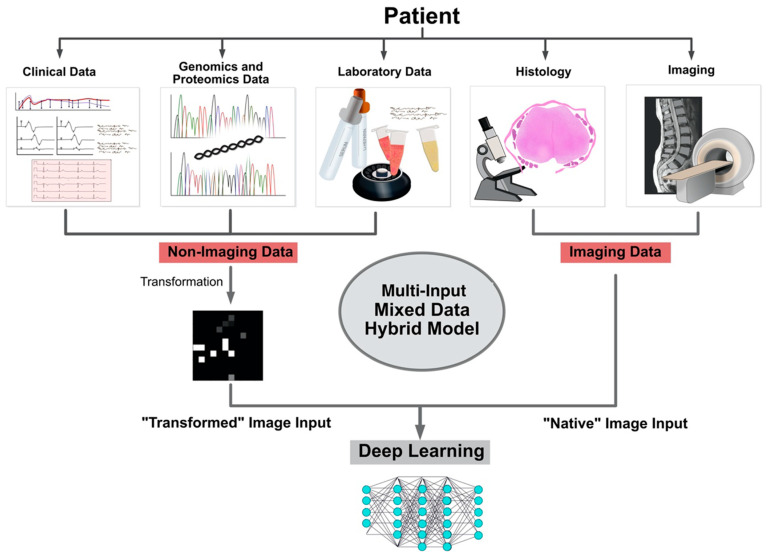
Illustration of multi-input mixed data model application in clinics.

The first applications performed on healthcare datasets were mainly focused on image data, such as the classification of skin cancer types, diabetic retinopathy, or pneumonitis in chest x-rays [53,54]. Multiple features in a dataset might contribute to a specific outcome of interest. These features are often not only relevant within a particular data type. A standard convolutional neural network architecture can consider multiple factors for prediction tasks while evaluating the impact of every factor on the target variable. Combining various data types for deep learning algorithms, which can require multiple inputs, can be seen as a multi-input mixed data deep learning approach based on a hybrid machine learning model pre-processing multiple data types. However, different data types can often not be processed within a single CNN. As discussed in the previous section, tabular data can be converted into image sets utilizing various techniques. The converted dataset can then be fed into the neural network via a separate input along with the non-converted dataset. The information can then be concatenated for feature processing and computing the prediction of these inputs. This approach is illustrated in Figure 2.

**Figure 3:**
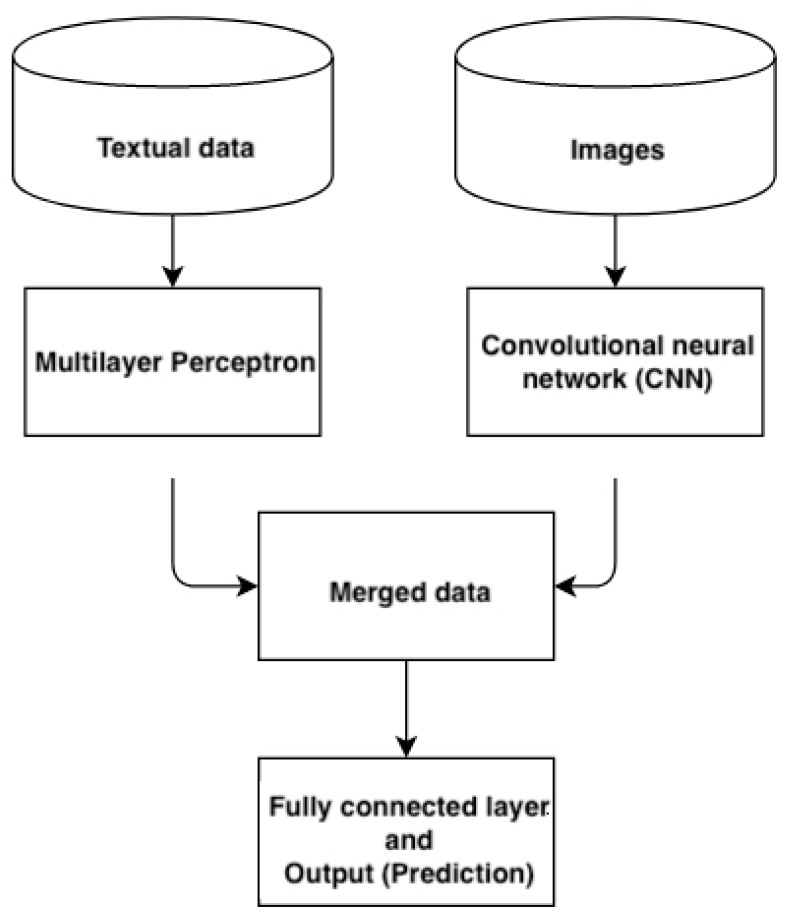
Illustration of multi-input mixed data architecture using two separate inputs, handled by a multilayer perceptron and a convolutional neural network before concatenation and outcome prediction.

The inputs used can be images, audio, text, and videos, for example. They can have multiple dimensions and are not restricted to specific variable types (e.g., continuous or categorical variables). This would allow surgeons to also implement text, audio, and videos from patients’ clinical data obtained during spine examinations in such models. Usually, the textual variables are normalized and scaled in pre-processing steps for better handling in the CNN. There are typically the following primary strategies for concatenating the multiple inputs [54]: 1. early concatenating strategies where the concatenation is performed in the input layer; 2. intermediate concatenating strategies, where the concatenation is also performed at the input layer, but backpropagation is used to propagate loss from the prediction model to the feature extracting networks; 3. concatenating strategies where the concatenation is performed at the output layers of different CNN branches. However, the pre-processing steps of converting the textual data into images for multi-input models are highly variable. The combination of both is currently not reported in the literature to the best of our knowledge. Textual inputs which have not been converted with techniques shown in the previous section could then be handled through a separate input along with the CNN input of the images. This can be, for example, done via Multilayer Perceptron, which handles one input before concatenation [55]. The Perceptron is able to solve classification tasks by the Stochastic Gradient Descent, which is used to minimize the distance between misclassified points and the decision boundary, and an activation function. Multilayer perceptron, a feedforward algorithm, can have multiple hidden layers and can also handle non-linear data. The structure of such an approach is shown in Figure 3.

It is also possible to construct random forests, support vector machines, and variations of CNN models which can handle multiple inputs. For example, Li et al. introduced a concatenation framework capable of handling various data types enabling shortcut connections to the fully-connected layer, which is then directly fed into the output layer for predictions [56]. They reported satisfying accuracies across multiple datasets. This would allow researchers to integrate clinical data, radiological images, and genetic architectures from patients obtained in diagnostic examinations. Additional efforts were made by authors combining deep learning models with the help of ensemble methods using ultrasound images and x-ray images as data inputs [57,58]. Vasile et al., for example, used x-ray images, symptoms, and clinical and biological variables within an ensemble of deep learning models to predict the severity of COVID-19 diagnosis [57]. In combination with imaging techniques such as in vivo or ex vivo reflectance confocal microscopy or ultrasound imaging, this multimodal approach could solve prediction tasks in real-time, such as chairside applications [52,59]. Furthermore, Yuan et al. recently introduced a general architecture for Hybrid deep neural networks supporting mixed inputs reporting that the Hybrid model reached higher accuracies with classical MLP and CNN models [60]. Notably, the majority of studies published to date using multi-input models applied early concatenating strategies [54]. The techniques applied in these studies ranged from simply concatenating image and clinical features [61,62,63], applying dimensionality reduction techniques before early concatenation [64], to medical image feature extraction using automated or manual feature extraction methods before concatenation with textual data [65,66,67]. Interestingly, the fourth possible strategy of converting textual data into images for feeding them into multi-input models is not published to date, to the best of our knowledge. Our workgroup is currently constructing a hybrid multi-input mixed data model that converts the textual data of spine surgery patients into image datasets before feeding them into the hybrid model. It would be highly interesting to evaluate whether this strategy would result in higher accuracy than using the tabular data or imaging data of patients alone. This would open the door for an algorithm that could be easily integrated into clinical software for supported decision-making.

## 4. Available Artificial Intelligence-Based Models and Classical Statistical Prediction Models Utilized in Spine Surgery

References in this section were researched in Pubmed (Medline) and Web of Science utilizing the following search terms connected by Boolean operators: (“spine surgery”) AND (“machine learning” OR “artificial intelligence”) using both “MeSH terms” and “All fields” searches. An additional search in Google Scholar was conducted for grey literature. Furthermore, reference lists of the extracted works were screened.

Artificial intelligence-based approaches in spine surgery can help to improve the accuracy currently reported using traditional statistical modeling. Our literature search, which aimed to find predictive studies (prediction of clinically relevant outcomes, such as PROMs, complications, and mortality) utilizing machine learning methods in spine research, revealed that most of the work on this topic has been completed within the last few years. A list of these spine surgery outcome prediction algorithms published to date is shown in Table 1. Seventeen studies predicted PROMs [68,69,70,71,72,73,74,75,76,77,78,79,80,81,82,83,84], fifteen predicted complications [85,86,87,88,89,90,91,92,93,94,95,96,97,98], seven examined the discharge disposition after spine surgery [99,100,101,102,103,104,105], seven predicted the length of hospital stay [81,87,92,102,105,106,107], six predicted readmission or re-herniation after spine surgery [100,108,109,110,111,112], four predicted mortality rates [113,114,115,116,117], three predicted prolonged opioid use after spine surgery [118,119,120], two predicted the need for blood transfusions [121,122], one focused on the duration until “return to work“ [123], one focused on 3D spinal alignment [124], and one predicted the future fracture probability after spine surgery [125]. Twenty-six of the studies included artificial neural networks within their prediction models, whereas thirty-seven utilized machine learning methods without the use of artificial neural networks. None of the studies applied multi-input mixed data models for prediction modelling. 

One of the most prominent applications of machine learning in spine surgery for prediction modeling was performed by Ogink et al. in 2019 [103]. They used the American College of Surgeons National Surgical Quality Improvement Program database to predict the non-home discharge after lumbar spinal stenosis surgery applying a convolutional neural network. They reported an area under the curve (a measure of accuracy in diagnostic tests, ranging from 0 (inaccurate) to 1 (perfect accuracy)) of 0.74, which was confirmed by another study validating the model [105]. Although they only used non-imaging data for their prediction, the results reveal the potential of machine learning approaches in predicting spine surgery outcomes. Another study published by Khan et al. in 2021 applied several machine-learning algorithms, including a classification tree, support vector machine, partial least squares, generalized boosted models, and multivariable adaptive regression splines to predict the health-related quality of life using the SF-36 form in patients who underwent surgery for degenerative cervical myelopathy. The results achieved an area under the curve of up to 0.78 for the multivariable adaptive regression spline, indicating a good degree of accuracy [74]. However, the sample size was small, with 130 samples for the training set. Moreover, no multi-input model was applied to handle patient data. Nevertheless, the approach reached considerable accuracy considering that tabular data was used solely. Varghese et al. reported an accuracy of 99% when using a random forest regression model to predict the pull-out strength of pedicle screws in osteoporotic and normal bone considering the density, insertion depth, and insertion angle feature variables [126]. This highlights that simple non-neural network-based modeling of simple tabular data can also lead to high accuracy in prediction tasks. 

Hoffman et al. applied traditional statistical techniques (multivariate linear regression) and machine learning techniques (support vector machines) to predict the Oswestry disability index (ODI) and the modified Japanese Orthopaedic Association questionnaire [71]. They reported that both outcome measures could be more accurately predicted using a support vector machine compared to the t-test statistical method. Support vector machines seem to be a well-performing approach in spine surgery when no imaging data are processed because it performs well in cases where the sample sizes are limited and the number of features is large [127]. However, the predictability may be a severe limitation in cases where the sample size is small. In these cases, the classical statistical approach might be better suited [128].

One advantage of deep learning techniques over simple machine learning techniques is that they allow for in-depth processing of imaging data, which can lead to more accurate predictions. Kim et al., for example, applied artificial neural networks to predict complications following posterior lumbar spine fusion [91]. They reported that artificial neural networks outperformed the American Society of Anesthesiology classification and logistic regression as a classical statistical technique in predictive accuracy for several complication types. Several open-access web applications have been introduced and allow surgeons to predict the prolonged opioid prescription [119,129], postoperative failure [98], in-hospital and postoperative mortality [113,114,116], and discharge disposition [101,103,104] using a simple online application platform, where predefined variables can be filled in. They allow for external validation and might be improved in the future to be more precise based on increasing data collection, which is currently being seen in spine surgery.

One of the main advantages of using deep learning algorithms is that training can be performed without predefining the variables and features that need to be included. This is especially advantageous for high-volume, multidimensional, and complex data types, including genomic or sequencing data, which require high computational resources and time-consuming annotations. These also require experts for feature selection or feature engineering [137]. However, images can also be taken in 3D, such as computed tomography scans or magnetic resonance images, and would need extensive pre-processing or complex strategies for multi-input processing [19]. One way to deal with such multi-slice data is to split them into multiple 2D images per patient or apply 3D neural networks for multi-input mixed-data hybrid data processing to overcome the applicable selection bias [138]. Notably, the generalizability of images is limited because the data are obtained through different methods (e.g., different MRI models), which might be particularly problematic when obtaining genomic data that are also dependent on the platform [139]. Especially for genomic data, for example, to classify cell types of intervertebral discs based on sequencing data, the large number of data points are better processed through more time-efficient methods. Combining the findings from such datasets with histopathological data can also be used for survival predictions during cancer research, which may also extend to many spinal diseases [140]. Furthermore, artificial intelligence can be applied to assess molecular markers of spine tumors, predicting the survival of primary spine tumors or metastatic recurrence rates [140]. In summary, the impressive amount of research performed on the applicability of artificial intelligence in clinically highly relevant prediction tasks is opening the door for a new kind of decision-making, which is currently on the rise in spine surgery.

## 5. Future Perspectives and Limitations

The combination of different datatypes allows for a multi-perspective view of patients’ data. This approach can reveal more information than the inclusion of one datatype alone. The clinical information is especially relevant for the interpretation of radiological images. Not having access to the laboratory or clinical data has been shown to significantly impact the interpretability of radiological images [141,142]. This was demonstrated in a survey where most radiologists reported that the availability of clinical information highly affected their reports [143]. Thus, this can also be assumed to be relevant for artificial intelligence models simulating human behavior. Notably, the volume of workload in surgery is very high. As such, when considering that spine surgeons often have to perform interpretation tasks during night shifts or at times of high workloads, the interpretation of multiple data types can be prone to errors. An automated assessment could help integrate an alert system for spine surgeons who can then take further care of their patients in cases whereby artificial intelligence indicates some sort of attention regarding the prediction of outcomes. 

The growth in the number of publications focusing on deep learning for images is enormous, whereas hybrid models are only just beginning to grow [54]. Several factors have impacted their development and implementation. These factors range from data-sharing limitations in healthcare institutions currently to the integration of machine learning algorithms in clinical settings considering the “good machine learning principles” [8]. It would be advantageous to consider these limitations before the implementation is planned. Following this strategy may allow healthcare specialists to learn from previously reported difficulties in the implementation phase. Consequently, the implementation will be more time-efficient and resource-oriented. Hence, the full potential of machine learning applications in healthcare settings can be maximized while avoiding problems that may arise due to the inherent privacy governance in healthcare [8].

Reliable data and algorithms are necessary but insufficient for implementing machine learning techniques in clinical settings. The application of machine learning requires comprehension and assessment of its implications to clinicians, patients, and other nodes in the healthcare system while developing the algorithms in real-time based on patient data in rapidly changing clinical environments. Increased collaboration among researchers and healthcare providers is needed regarding the development of machine learning workflow and data training, with outputs reflecting the needs of patients, to ensure that these systems are feasible, trustworthy, and usable in clinical settings. Providing datasets in repositories is highly encouraged. However, even if data deposition is made mandatory for research performed in spine surgery, there are several challenges to making the data in these repositories useful for machine learning and deep learning tasks. One must obtain ethics approval which, depending on the relevant government policies, may be a somewhat complicated process. Then, proper and careful labeling of the data is a critical task that can limit usability when not performed in a standardized way by experts [144]. As such, in cases whereby the researcher could not validate the labeling, the “failure” in data labeling and annotations would still exist in the database.

Furthermore, imaging plays a crucial role in spine surgery; however, processing 3D images such as MRI and CT can be very time-consuming. In addition, different machines used to obtain the images might introduce selection bias [145]. Consequently, this can affect the translation of data to other institutes. In this case, it is particularly warranted to provide multi-center data in such repositories or combine similar datasets from different institutes utilizing standardized techniques. The more data the algorithm processes, the more applicable it can be regarding unseen data. Notably, unsupervised machine learning techniques do not need any labeling of data [146]. Instead, these techniques identify patterns in the dataset through dimension reduction techniques and are mainly exploratory. However, they still do not perform well on non-qualitative small datasets and require more computational power than large datasets. Overall, they can be more complex than supervised learning methods and are mainly intended for clustering tasks, such as disease taxonomy, based on their pathophysiology [147]. In this case, the validation is limited as there are no labels and, therefore, no “ground truths” to confirm the obtained results of the task performed by the unsupervised learning algorithm [148]. Thus, experts need to validate the performance afterward, which can also be time-consuming. 

Finally, data in spine surgery is collected at an institutional level. The anonymization and transfer of large datasets might require specialist infrastructure, along with a dedicated team of data scientists to handle such large data volumes [149] Implementation of repositories that lower the barriers for large volume data transfer, such as 3D images from clinics to the database without compromising data privacy, might increase the efficiency of data transfer and thus could help increase the amount of data provided by surgeons [26].

Furthermore, researchers developing machine learning algorithms must also consider model updates when larger or novel datasets are made publicly available or if algorithms need to be improved based on more recent research. As a result, continuous monitoring of machine learning applications becomes a necessity. This can result in high maintenance costs and can also be time-consuming. Failures to comply can have far-reaching consequences for the patients. After implementing such algorithms, continuous monitoring can ensure that the algorithms are working as expected, disregarding the data type that is used. However, as in other automated applications, such as self-driving cars, the applications should not be the only pillar decisions are based on [150,151]. Clinicians should consider these applications as decision-supporting tools, which should be questioned every time they are applied. Finally, the workflow should be explainable to maintain safety, which is particularly complicated in complex machine learning models involving artificial neural networks. While these uprising decision-supporting techniques can add significant value to healthcare systems, they still have substantial challenges that need to be considered by all clinicians aiming to implement these in their daily practices. Poorly implemented tools that generate unnecessary alerts can not only be a threat to patients and lead to worse outcomes, but they could also significantly increase healthcare costs. 

Furthermore, accurate models require large data sets to sufficiently perform in various clinical environments and enable them to generalize well to new and unseen data [36]. As such, conclusions drawn from machine learning in healthcare applications are dependent on the quality of the training dataset with which the algorithm was initially trained. Hence, the deposition of research data can be considered an essential step in research as this ensures that models can be tested against new unseen datasets for external validation before evaluating whether the applications prove themself in clinical settings. As computer technologies develop, machine learning processes can be tested against a vast amount of high-quality medical data to evaluate outcome predictions. While the application of machine learning in healthcare is still in an early phase, the lack of data preventing its integration and implementation into the health care system might slow down the development process. Encouraging data deposition by researchers helps to take advantage of such algorithms towards patient care in the near future. Hence, we encourage researchers to make their data available publicly, particularly when the data set is multimodal, considering that such datasets are currently scarce regarding spine surgery specifically. This severely limits the progress of machine learning implementation in spine surgery.

## 6. Conclusions

As artificial-intelligence-based decision-making tools develop, the availability of databases and modern hybrid algorithms can accomplish intricate and highly complex tasks to help clinicians with their daily practice. The various models published to date can assist spine surgeons in predicting the outcomes of treatments, estimating the probability of failure, and detecting disease patterns in multimodal data. Implementing “good machine learning principles” and strengthening cooperation between healthcare providers and industries will be beneficial for the use of modern machine learning algorithms in clinics. The integration of multimodal data in novel machine learning hybrid models might better reflect patient information, and more research in this field is highly warranted. Data repositories containing data from different institutes can help researchers develop such algorithms better. Various techniques, including Federated Learning and crowdsourcing, can be beneficial for an unbiased implementation. However, although these algorithms might be developed using large-scale data, they need to be still questioned and only considered as decision-supporting tools in clinics. Continuous updates are necessary that integrate new data and research into existing algorithms. Following these recommendations could hasten the development process and lead to a safer integration of artificial intelligence in healthcare environments. This narrative literature review highlighted the benefits of machine learning implementations in spine surgery, focusing on multimodal data. The findings reveal promising prior research to develop multi-input mixed-data hybrid decision-supporting models. As such, their implementation into clinics seems to be only a matter of time.

## Figures and Tables

**Table 1:** List of spine surgery outcome prediction studies. ANN: artificial neural networks; DTL: decision tree learning; LOR: logistic regression; elastic-net-LOR: elastic-net penalized logistic regression; MLOR: multivariate logistic regression; MLIR: multivariate linear regression; MARS: multivariable adaptive regression splines; MLP: multilayer perceptron; LASSO: least absolute shrinkage and selection operator; k-NN: k-nearest neighbor; BN: Bayesian network; EL: ensemble learning; NB: Naïve Bayes; RF: random forest; GLM: generalized linear model; GLMnet: elastic-net generalized linear model; GBM: gradient boosting machine; GAM: generalized additive model; PLS: partial least squares; XGBoost: extreme gradient boosting; NLP: natural language processing; pLDA: penalized linear discriminant analysis; RBF: radial basis function network; SVM: support vector machine; SVR: support vector regression; SGB: stochastic gradient boost; PROMs: patient-reported outcome measures; LOS: length of hospital stayS.

Author (Year)	Number of Datapoints	Algorithm	Intervention/Diagnosis	Outcome
Aldebeyan et al. (2016) [99]	15.092	MLOR	lumbar spine fusion surgery	discharge disposition
Andre et al. (2020) [85]	60	ANN	lumbar decompression	complications
Arvind et al. (2018) [86]	20.879	ANN, LOR, RF, SVM	cervical discectomy and fusion	complications
Babaee et al. (2018) [69]	480	MLP, RBF, LOR	posterior spinal fusion surgery	PROMs
Bekelis et al. (2014) [87]	2732	MLOR	corpectomy; spinal fusion	complications; LOS
Berjano et al. (2021) [68]	1243	RF	spinal lumbar arthrodesis	PROMs
Dong et al. (2021) [121]	152	SVM, DTL, MLP, NB, k-NN, RF	spinal fusion	blood transfusion
Durand et al. (2018) [122]	1029	RF, DTL	spinal deformity	blood transfusion
Finkelstein et al. (2021) [70]	122	LASSO, bootstrapping	spinal decompression/fusion surgery	PROMs
Goyal et al. (2019) [100]	59.145	ANN, GLM, GLMnet, GBM, NB, pLDA	spinal fusion	discharge disposition; readmission
Han et al. (2019) [88]	1.106.234	LASSO-R; LOR	spine surgery (various diagnoses and procedures)	complications
Harada et al. (2021) [130]	2630	XGBoost	lumbar microdiscectomy	disc re-herniation
Hoffmann et al. (2015) [71]	27	MLIR; SVR	cervical spondylotic myelopathy	PROMs
Hopkins et al. (2019) [108]	23.263	ANN	spinal fusion surgery	30-day hospital readmission
Hu et al. (2022) [118]	1316	SORG-algorithm (SGB)	lumbar disc herniation	prolonged postoperative opioid prescription
Janssen et al. (2021) [72]	77	RF	lumbar spinal fusion	PROMs
Kalagara et al. (2018) [109]	26.869	GBM	lumbar laminectomy	readmission
Karhade et al. (2019) [131]	2737	ANN, elastic-net-LOR, SGB, SVM, RF	cervical discectomy and fusion	sustained opioid prescription
Karhade et al. (2018) [101]	26.364	ANN, BN, DTL, SVM	lumbar disc surgery	discharge disposition
Karhade et al. (2019) [115]	1053	ANN, elastic-net-LOR, SGB, SVM, RF	spinal epidural abscess	in-hospital and 90-day post-charge mortality
Karhade et al. (2019) [114]	1790	ANN, DTL, BN, SVM	spinal metastasis surgery	30-day-mortality
Karhade et al. (2019) [113]	732	ANN, elastic-net-LOR, SGB, SVM, RF	spinal metastatic disease management	90-day-mortality and 1-year-mortality
Karnuta et al. (2019) [102]	3807	NB	lumbar spinal fusion	discharge disposition and LOS
Karhade et al. (2021) [89]	1035	XGBoost (NLP algorithm)	anterior lumbar spine surgery	complications
Karhade et al. (2022) [110]	708	XGBoost (NLP algorithm)	posterior lumbar fusion	readmission
Khan et al. (2021) [74]	193	SVM, GAM (LogitBoost), MARS (earth), GBM, DTL, RF, LOR, PLS	degenerative cervical myelopathy	PROMs
Khan et al. (2021) [73]	757	SVM, GAM (LogitBoost), MARS (earth), GBM, DTL, RF, LOR, PLS	degenerative cervical myelopathy	PROMs
Khor et al. (2018) [75]	1583	MLOR	lumbar spine surgery	PROMs
Kim et al. (2018) [90]	4073	ANN, LOR	adult spinal deformity	complications
Kim et al. (2018) [91]	22.629	ANN, LOR	lumbar spine fusion	complications
Kuo et al. (2018) [127]	532	ANN, SVM, DTL, BN	spinal fusion	cost prediction
Kuris et al. (2021) [111]	63.533	ANN	posterior lumbar interbody fusion	readmission
Lewandrowski et al. (2020) [76]	383	ANN, LOR	lumbar spinal decompression	PROMs
Li et al. (2021) [132]	385	LOR, GBM, XGBoost, RF, DTL, MLP	osteoporotic vertebral compression fracture	bone cement leakage
Maki et al. (2021) [133]	478	GBM, XGBoost, RF, LOR	cervical ossification of the posterior longitudinal ligament	PROMs
Massaad et al. (2022) [92]	484	k-means clustering analysis, LOR	spinal metastases surgery	complications, LOS, mortality
McGirt et al. (2015) [77]	1803	BN, LOR	lumbar spine surgery	PROMs
Merali et al. (2019) [78]	757	ANN, LOR, DTL, RF, SVM	degenerative cervical myelopathy	PROMs
Nunes et al. (2022) [112]	215.999	ANN, Cox-Regression, XGBoost, DTL, NB, RF	thoracolumbar fractures	30-day readmission
Ogink et al. (2019) [104]	9338	ANN, DTL, BN, SVM	Spondylolisthesis	discharge disposition
Ogink et al. (2019) [103]	28.600	ANN, DTL, BN, SVM	lumbar spinal stenosis	discharge disposition
Oh et al. (2017) [79]	234	DTL	adult spinal deformity	PROMs
Papić et al. (2016) [123]	153	DTL, SVM, MLP	lumbar microdiscectomy	return to work
Pasha et al. (2021) [124]	371	EL	adult idiopathic scoliosis	3D spinal alignment
Passias et al. (2018) [134]	101	DTL	cervical deformity surgery	distal junctional kyphosis
Pedersen et al. (2020) [80]	1968	ANN, DTL, RF, SVM	lumbar disc herniation	PROMs
Ratliff et al. (2016) [93]	279.135	LASSO, LOR	spine surgery (various diagnoses and procedures)	complications
Russo et al. (2021) [106]	1516	MLOR, LASSO	cervical discectomy	LOS
Shah et al. (2019) [98]	367	ANN, RF, SVM, SGB, elastic-net-LOR	spinal epidural abscess	complications
Shah et al. (2021) [116]	298	SORG-algorithm (SGB)	spinal metastasis surgery	90-day and 1-year mortality
Shah et al. (2021) [94]	6822	LOR, RF, GBM, XGBoost	posterior cervical spinal fusion	complications
Siccoli et al. (2019) [81]	635	ANN, RF, XGBoost, DTL, GLM, k-NN	lumbar spinal stenosis	PROMs, reoperations, LOS
Staartjes et al. (2019) [82]	422	ANN, LOR	lumbar discectomy	PROMs
Staartjes et al. (2022) [83]	817	GLM, elastic-net-LOR, k-NN	lumbar spinal fusion	PROMs
Stopa et al. (2019) [105]	144	ANN	lumbar disc surgery	discharge disposition, LOS
Veeramani et al. (2022) [95]	54.502	ANN, LOR, MVR, DTL, RF, GBM, XGBoost	anterior cervical discectomy and fusion	complications
de Vries et al. (2021) [125]	7578	ANN, RF, Cox-regression	fracture patients with osteopenia and osteoporosis	future fracture
Wang et al. (2021) [96]	13.500	XGBoost	posterior lumbar fusion	complications
Wang et al. (2021) [135]	184	SVM	posterior laminectomy and fusion with cervical myelopathy	complications
Wong et al. (2020) [136]	1164	SVM	anterior cervical discectomy and fusion	complications
Wirries et al. (2021) [84]	60	ANN	lumbar disc herniation	PROMs
Yang et al. (2021) [117]	427	SORG-algorithm (SGB)	spinal metastasis surgery	90-day and 1-year mortality
Zhang et al. (2021) [107]	1281	LOR, DTL, RF, XGBoost, GM	spinal fusion surgery	LOS
Zhang et al. (2020) [120]	19.317	ANN, LASSO, LOR, RF, SGB	thoracic or lumbar spine surgery (low back pain)	prolonged opioid use
